# A Novel Therapeutic Approach Targeting Spinal Accessory and Dorsal Scapular Nerves for the Relief of Posterior Neck, Trapezius, and Interscapular Pain

**DOI:** 10.3390/jcm13247754

**Published:** 2024-12-19

**Authors:** Sin-Hye Park, Sin-Hwe Kim, Minha Kim, Jong Burm Jung, Kwangwoon Choi, Daewook Lee, Je-Hun Lee, Jeong Won Seong, Cheol-Jung Yang

**Affiliations:** 1 Department of Food Science and Nutrition, Korean Institute of Nutrition, Hallym University, Chuncheon 24252, Republic of Korea; shpark88@hallym.ac.kr; 2 Department of General Surgery, Borntouch Orthopaedic Clinic, Seoul 05269, Republic of Korea; sinhwekim@gmail.com; 3 Department of Orthopedic Clinic, Borntouch Orthopaedic Clinic, Seoul 05269, Republic of Korea; 507mhk@gmail.com; 4 Department of Rehabilitation Medicine, Borntouch Orthopaedic Clinic, Seoul 05269, Republic of Korea; huk0713@naver.com; 5 Department of Orthopedic Surgery, Borntouch Orthopaedic Clinic, Seoul 05269, Republic of Korea; memeeda@gmail.com; 6 Department of Anesthesiology and Pain Medicine, Gwangju Wooridul Hospital, Gwangju 61963, Republic of Korea; rason747@gmail.com; 7 Department of Sports, Korea National Sport University, Seoul 05541, Republic of Korea; leejehun@knsu.ac.kr; 8 Korea Institute for Applied Anatomy, College of Sports Science, Korea National Sport University, Seoul 05541, Republic of Korea; 9 Department of Family Medicine, Borntouch Orthopaedic Clinic, Seoul 05269, Republic of Korea

**Keywords:** posterior neck pain, trapezius pain, interscapular pain, spinal accessory nerve, dorsal scapular nerve, nerve entrapment point

## Abstract

**Background/Objectives:** Posterior neck, trapezius, and interscapular pain, exacerbated by poor posture such as forward head and rounded shoulders, is common. In this study, we aimed to assess the clinical outcomes of isotonic saline injections at nerve entrapment points (NEPs) within the sternocleidomastoid (SCM) and scalenus medius (SM) muscles for alleviating spinal accessory nerve (SAN) and dorsal scapular nerve (DSN) compression in patients suffering from posterior neck, trapezius, and interscapular pain. **Methods:** In this retrospective study, 68 patients were included, with 34 receiving isotonic saline injections and 34 undergoing Extracorporeal Shock Wave Therapy (ESWT) as a control. The clinical outcomes were evaluated using the Visual Analog Scale (VAS) and Percent Pain Intensity Difference (PPID) before and after therapy. The effectiveness of isotonic saline injections targeting NEPs in the SCM and SM muscles in relieving pain associated with SAN and DSN entrapment was assessed. **Results:** Both treatments significantly reduced VAS and PPID scores, with injection therapy showing a larger treatment effect size (Cohen’s d: 3.521 for VAS and 3.521 for PPID) compared to ESWT (Cohen’s d: 1.379 for VAS and 1.710 for PPID). The mean clinically important difference observed for VAS was 4.2, exceeding the expected value of 2.6, indicating a substantial improvement in pain and patient quality of life. **Conclusions:** Isotonic saline injections at the NEPs of SAN and DSN within the SCM and SM muscles might potentially reduce posterior neck, trapezius, and interscapular pain associated with possible nerve entrapment, without causing complications. Further research is needed to validate these findings in larger, controlled trials.

## 1. Introduction

Posterior neck, trapezius, and interscapular pain are widespread, with prevalence rates varying from 5.9% to 38.7% in the general population [1]. Neck pain is notably prevalent among athletes, with occurrence rates ranging from 8% to 45% in a single week period, 38% to 73% annually, and approximately 48% over a lifetime [2]. The widespread use of mobile devices and computers has led individuals to adopt poor postures, such as forward head posture and rounded shoulder posture, which result in prolonged periods of looking down at screens [3,4]. Moreover, a prior study demonstrated the significant prevalence of forward head posture among young adults afflicted with myopia [5]. These postures contribute to excessive strain on the erector spinae, rhomboids, levator scapulae, and trapezius muscles, commonly leading to posterior neck, trapezius, and interscapular pain [6,7,8].

Some studies have highlighted the role of electromyographic analysis in understanding muscle activity related to neck pain [9,10]. These studies investigated electromyographic activity differences in individuals with and without neck pain, focusing on the upper trapezius muscle during prolonged writing tasks and cervical muscle profiles, respectively. These findings underscore the significance of detailed electromyographic assessments in diagnosing and managing neck pain conditions.

Seong defined a nerve entrapment point (NEP) as a location where a muscle physically compresses a nerve, causing pain in the nerve-innervated region [11]. Poor postures, such as forward head posture and rounded shoulder posture, cause overstrain in muscles supporting the cervical region, including the sternocleidomastoid (SCM) and scalenus medius (SM) muscles [3,4]. Consequently, the spinal accessory nerve (SAN) passing through the upper 1/3 of the SCM and the dorsal scapular nerve (DSN) passing through the mid-portion of the SM can become compressed [12,13]. This entrapment may lead to ischemic pain in the posterior neck, trapezius, and interscapular area [11].

Several studies have revealed various treatment options for managing posterior neck, trapezius, and interscapular pain. Common pharmacological interventions, including nonsteroidal anti-inflammatory drugs, muscle relaxants, and analgesics, have been identified as effective approaches. Physical therapy, incorporating exercises targeting specific muscle groups, stretching, and posture correction, plays a crucial role in the treatment of neck, shoulder, and back pain [14,15]. Additionally, the effects of alternative treatments such as dry needling, massage therapy, trigger point injection, and Extracorporeal Shock Wave Therapy (ESWT) have been reported [16,17]. Lidocaine injection therapy within the intramuscular innervation zones of the trapezius muscle effectively reduces chronic neck pain, especially when targeting both the mid-upper and lower trapezius zone for myofascial pain syndrome [16]. Moreover, dry needling provides effective short- and medium-term relief for myofascial trigger point (MTrP) pain in the neck and shoulders [17]. Additionally, consecutive sessions of ESWT effectively treat neck and shoulder pain syndrome, improving function and reducing pain. Particularly, receiving ESWT twice per week has been shown to be effective in simultaneously reducing pain and tenderness [18].

To our knowledge, no studies have specifically addressed treating entrapment of the SAN and DSN within SCM and SM muscles. The objective of this study is to analyze the clinical effects of isotonic saline injection at identified NEPs located in the upper 1/3 of SCM and mid-portion of SM, which are believed to be associated with SAN and DSN entrapment. In addition, we included an ESWT group as a control to compare the effectiveness of treatment modalities. It is hypothesized that administering injection therapy at these points to release entrapped nerves may effectively relieve posterior neck, trapezius, and interscapular pain, and this effect was compared with the outcomes observed in the ESWT group.

## 2. Materials and Methods

### 2.1. Study Design and Patients

**Figure 1:**
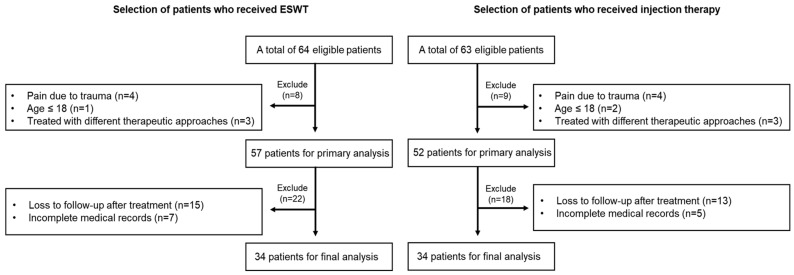
Flowchart of the patient selection process.

From 1 November 2023 to 31 December 2023, patients who presented with posterior neck, trapezius, and interscapular pain and who received either injection therapy or ESWT at Borntouch Orthopedic Clinic were retrospectively selected through chart review. For patients who received ESWT, a total of 64 eligible patients were initially considered for inclusion in this study. The primary treatment strategy was determined by random allocation. Patients were randomly assigned to receive either ESWT or injection therapy during the study period, ensuring that the allocation of treatments was unbiased. The randomization process was carried out to ensure an equal distribution of patients across both treatment groups, thus maintaining consistency and reducing potential bias in treatment allocation. The exclusion criteria included the following: pain resulting from trauma (n = 4), individuals under the age of 18 (n = 1), and those treated with different therapeutic approaches (n = 3). Additionally, patients with loss to follow-up after treatment or incomplete medical records were excluded from the final analysis. After applying these criteria, 34 patients were eligible for analysis in this study (Figure 1). For patients who received injection therapy, out of a total of 63 patients, those patients were excluded based on the following criteria: trauma such as a traffic accident (n = 4), age under 18 (n = 2), those who received other therapeutic approaches (n = 3), history of neck surgery (n = 2), loss to follow-up (n = 13), and incomplete medical records (n = 5). Therefore, 34 patients were included in the final analysis (Figure 1).

### 2.2. Research Ethics

This study involved a retrospective chart review and was exempted from Institutional Review Board approval by the Public Institutional Review Board designated by the Ministry of Health and Welfare (P01-202401-01-036).

### 2.3. Extracorporeal Shock Wave Theraphy (ESWT) Procedure

ESWT was performed using a Wolf PiezoWave2 device (Richard Wolf GmbH, Knittlingen, Germany) to deliver focused shock waves to the affected areas of the posterior neck, trapezius, and rhomboid muscles. Patients were positioned in a prone position to allow for optimal access to the posterior neck, trapezius, and rhomboid regions. Key tender points were identified through manual palpation and patient feedback, focusing on areas of highest tenderness within the posterior neck, trapezius, and rhomboid muscles. The ESWT was then applied to these identified points, delivering 2500 pulses per session at a frequency of 8–12 Hz and an energy flux density of 0.1–0.3 mJ/mm^2^, depending on patient tolerance, to ensure both therapeutic effectiveness and comfort. Each treatment session was approximately 15 min in duration, and patients underwent treatment twice weekly for a period of four weeks.

### 2.4. Isotonic Saline Injection Techniques

**Figure 2:**
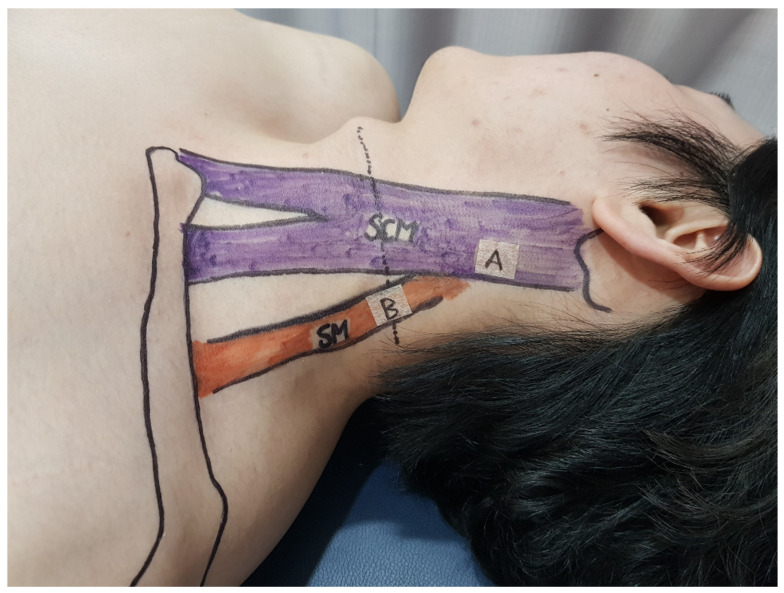
Observation of sternocleidomastoid (SCM) muscle tension. In this procedure, the patient was positioned lying down on the bed. To initiate observation, the patient’s head was turned 45 degrees away from the side exhibiting symptoms. Upon the practitioner’s instruction, the patient attempted to lift their head from the bed. This action is critical for detailed observation of the SCM muscle’s reaction, specifically when looking for signs of tense overextension. This figure captures the moment of the attempted head lift, highlighting the SCM muscle’s tense overextension, which is a key observation point for clinical assessment.

The patient was positioned lying down on the bed, with their head turned 45 degrees away from the affected side. The practitioner asked the patient to attempt to lift their head off the bed to observe the overextension of the SCM muscle (Figure 2).

#### 2.4.1. SAN Entrapment Point Injection Technique

**Figure 3:**
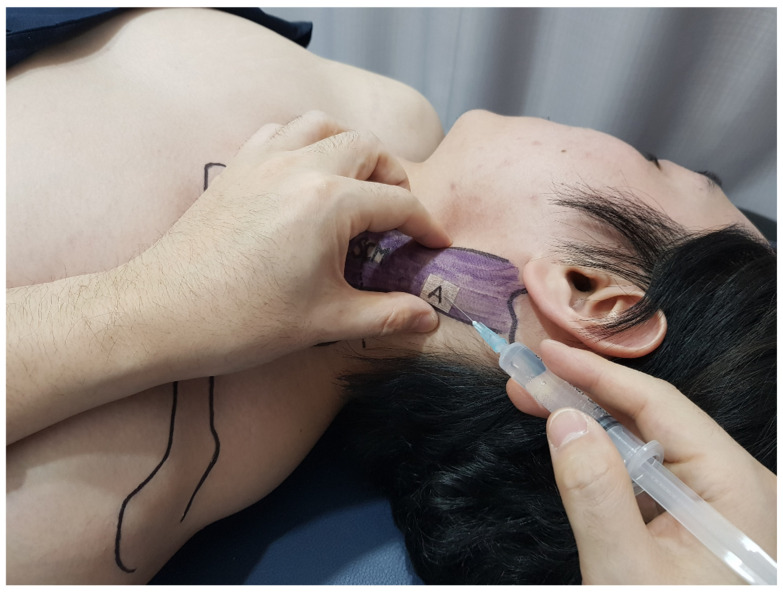
Spinal accessory nerve (SAN) entrapment point injection technique. This figure demonstrates the precise technique for administering an injection to alleviate symptoms associated with SAN entrapment. The practitioner used their left hand to apply pressure and tighten the upper 1/3 of the SCM muscle outwardly. This manipulation is crucial for delineating the injection site (A) and ensuring accurate needle placement. Using a 23-gauge, 1-inch needle syringe, the practitioner inserted the needle into the previously identified upper 1/3 point of the SCM, and isotonic saline was injected into the site (A). This procedure is depicted at the moment of injection, showing the precision of the technique. Abbreviations: SCM: sternocleidomastoid.

Using the thumb and index finger of the left hand, pressure was applied to tighten the upper 1/3 portion of the SCM muscle outwardly. Once this was complete, the patient’s head was lowered. The injection site was then sterilized with a topical alcohol solution. A 23-gauge, 1-inch needle syringe (Sungshim Medical Co., Bucheon, Republic of Korea) was inserted into the upper 1/3 point of the SCM muscle, and 4 mL of an isotonic saline solution was injected (Figure 3).

#### 2.4.2. DSN Entrapment Point Injection Technique

**Figure 4:**
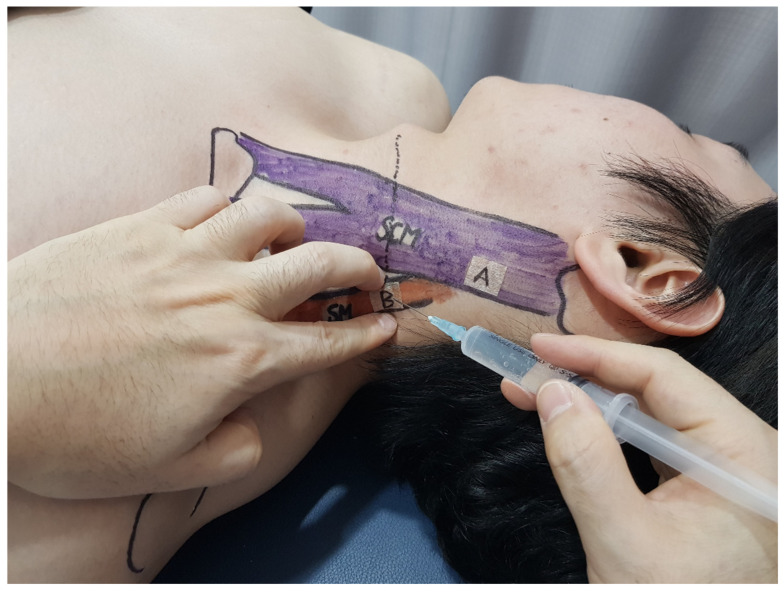
Dorsal scapular nerve (DSN) entrapment point injection technique. This figure demonstrates the precise technique for administering an injection to alleviate symptoms associated with DSN entrapment. The practitioner began by positioning the mid-portion of the SM muscle between the index and middle fingers of the left hand. This manipulation is crucial for delineating the injection site and ensuring accurate needle placement. Using a 23-gauge, 1-inch needle syringe, the practitioner inserted the needle into the previously identified mid-portion of the SM muscle, and isotonic saline was injected into the site. This procedure is depicted at the moment of injection, showing the precision of the technique. Abbreviations: SM, scalenus medius.

The Adam’s apple, or the point where the upper border of the clavicle and the lower tip of the mastoid process intersect with the larynx, was located. At this intersection point, the bulging SM muscle situated below the lower border of the SCM muscle was palpated. The index and middle fingers of the left hand were used to hold the SM muscle. After sterilizing the injection site with a topical alcohol solution, a 23-gauge, 1-inch needle syringe was inserted into the mid-portion of the SM muscle, and 4 mL of the isotonic saline solution was injected (Figure 4).

### 2.5. Outcome Assessment

The clinical evaluation utilized the Visual Analogue Scale (VAS) and Percent Pain Intensity Difference (PPID) for assessment [19,20]. The VAS is a subjective tool for measuring the intensity or severity of a specific symptom, typically pain. It consists of a straight line, usually 10 cm long, with endpoints that represent the extreme limits of the pain experience (e.g., “no pain” to “worst pain imaginable”). The PPID is a subjective tool for calculating the percentage reduction in pain relative to the baseline. In this approach, the baseline is initially set at 100 before treatment, serving as a reference point. The numeric value then represents the degree of improvement as a percentage.

**Figure 5:**
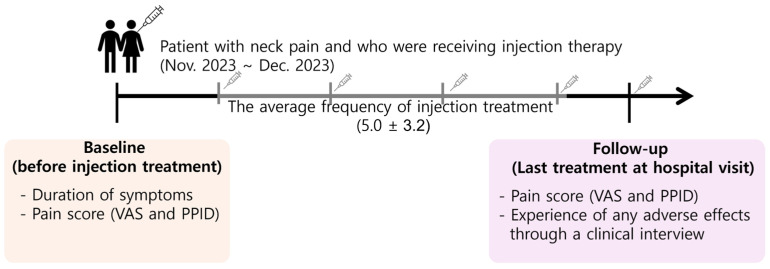
Graphical diagram of timeline and examination details of the injection treatment. This diagram illustrates the follow-up period for a study on injection treatment. At the baseline (before injection treatment), the data collected include the duration of symptoms and pain scores measured using the VAS and PPID. The average frequency of injection treatment over the course of this study was noted. At the follow-up, which occurred at the last hospital visit, the pain score was again measured using VAS and PPID, along with the recording of any adverse effects experienced by patients, assessed through a clinical interview. This timeline ensures consistent evaluation of patient outcomes before and after the treatment period. Abbreviations: VAS, Visual Analogue Scale; PPID, Percent Pain Intensity Difference.

The pain scores were evaluated before each injection or ESWT treatment, establishing a baseline, and during follow-up visits to assess the effects of the treatment. The outcome was determined based on the scores recorded on the last visit. Patients were also asked to report any adverse effects experienced during the treatment at each visit. The flow of treatment over time was presented as a simplified diagram, in the case of injection therapy (Figure 5).

### 2.6. Statistical Analysis

The sample size for this study was determined using a two-sided hypothesis testing approach, considering an anticipated effect size of a 20% reduction in VAS scores, a level of significance of 0.05, and a desired power of 0.90. The standard deviation was estimated at 20 based on data from a previous similar study [21]. As a result, the minimum required sample size for this study was calculated to be 25 patients. Nonetheless, a total of 66 patients were targeted for enrollment to ensure sufficient statistical power and account for potential dropouts.

Clinical outcomes, specifically VAS and PPID scores before treatment or injection and at the final follow-up, were compared using a paired *t*-test. Furthermore, to identify a clinical implication with the effect size of pain score differences between before and after ESWT treatment or injection therapy within the same group, we used the method of calculating the effect size, Cohen’s d [22]. The effect size was computed using the formula: Cohen’s d = Mean difference/standard deviation of difference. The interpretation of Cohen’s d follows these thresholds: (1) small effect: *d* = 0.2; (2) medium effect: *d* = 0.5; (3) large effect: *d* = 0.8. Statistical analyses were performed using SAS 9.4 software (SAS Institute, Inc., Cary, NC, USA).

## 3. Results

### 3.1. Patient Characteristics

In this retrospective study, a total of 68 patients were included, with 34 patients in the ESWT group and 34 in the injection therapy group. Table 1 describes the characteristics of patients in the ESWT and injection therapy treatment group at baseline. The average age of the patients was similar between groups, with a mean of 44.9 ± 11.7 years in the ESWT group and 44.2 ± 15.2 years in the injection group (*p* = 0.824). Gender distribution was also comparable between groups (*p* = 0.625). The duration of symptoms prior to treatment was 135.9 ± 379.3 days in the ESWT group and 172.1 ± 342.5 days in the injection group, with no statistically significant difference between groups (*p* = 0.681). The initial VAS scores, which measure pain intensity, were 5.5 ± 1.6 in the ESWT group and 6.2 ± 1.3 in the injection group, showing a trend toward higher initial pain levels in the injection group, although this difference did not show statistical significance (*p* = 0.058). Regarding treatment duration, patients in the ESWT group underwent treatment for an average of 16.1 ± 21.2 days, compared to 24.1 ± 22.7 days in the injection group, with no statistically significant difference observed (*p* = 0.138). The frequency of injections was similar between groups with no statistically significant difference (*p* = 0.060).

### 3.2. Effectiveness of ESWT and Injection Therapy on Pain Scores

Table 2 presents a comparison of changes in VAS and PPID scores between patients who received ESWT and those who received injection therapy. For the VAS score, baseline pain levels were slightly higher in the injection group (6.2 ± 1.3) compared to the ESWT group (5.5 ± 1.6), although this difference was not statistically significant (*p* = 0.058). At the final follow-up, the injection group had a significantly lower mean VAS score (2.0 ± 1.0) compared to the ESWT group (2.8 ± 1.9), with a *p*-value of 0.038, indicating a greater reduction in pain levels for the injection group. The change in VAS score from baseline to final follow-up was significantly larger in the injection group (4.2 ± 1.2) compared to the ESWT group (2.7 ± 2.0), with a *p*-value of < 0.001. Within each group, the reduction in VAS score from baseline to follow-up was statistically significant (*p* < 0.001), showing that both treatments led to significant improvements in pain.

For the PPID score, both groups started with the same baseline score of 100. By the final follow-up, the mean PPID score was significantly lower in the injection group (33.5 ± 17.7) compared to the ESWT group (50.0 ± 29.2), with a *p*-value of 0.007, indicating greater improvement in pain intensity for the injection group. The changes in PPID score from baseline to final follow-up were also significantly larger in the injection group (66.5 ± 17.7) compared to the ESWT group (50.0 ± 29.2), with a *p*-value of 0.007. Within both groups, reductions in PPID scores were statistically significant (*p* < 0.001), demonstrating significant pain relief from both treatments.

In summary, while both ESWT and injection therapy resulted in significant improvements in VAS and PPID scores, the injection therapy group showed a statistically significant greater reduction in pain intensity compared to the ESWT group.

### 3.3. Impact of ESWT and Injection Therapy on VAS and PPID Scores

Table 3 summarizes the effect sizes of treatment-induced changes in VAS and PPID scores for patients treated with ESWT and injection therapy. For the VAS score, the mean difference in pain reduction from before to after treatment was 4.177 in the injection group, compared to 2.706 in the ESWT group. The standard deviation of the difference was lower in the injection group (1.186) than in the ESWT group (1.962). The calculated *t*-values were 8.040 for ESWT and 20.530 for the injection group, both of which were statistically significant (*p* < 0.001), indicating substantial pain reduction in each group. The effect size, as measured by Cohen’s d, was significantly higher in the injection group (*d* = 3.521) than in the ESWT group (*d* = 1.379), suggesting a larger effect of the injection treatment on pain reduction as measured by VAS scores.

For the PPID score, the mean reduction from baseline was greater in the injection group (66.471) compared to the ESWT group (50.000). The standard deviation of difference was also lower in the injection group (17.732) relative to the ESWT group (29.233). The *t*-values were 9.970 for ESWT and 21.860 for injection therapy, with both values being of statistical significance (*p* < 0.001), confirming significant pain intensity improvement in both groups. Cohen’s d for PPID score reduction was *d* = 3.521 in the injection group and *d* = 1.710 in the ESWT group, again demonstrating a larger effect size for injection therapy.

In summary, both ESWT and injection therapy produced statistically significant reductions in VAS and PPID scores. However, injection therapy showed a consistently larger effect size for both pain measures, indicating greater treatment impact on pain reduction compared to ESWT.

In this study comparing ESWT and injection therapy for pain management, both treatments significantly improved VAS and PPID scores, indicating effective pain relief. However, injection therapy consistently showed a greater reduction in both VAS and PPID scores at the final follow-up, with a larger effect size for both pain measures compared to ESWT. This suggests that while both treatments are beneficial, injection therapy may have a more substantial impact on reducing pain intensity.

## 4. Discussion

This study demonstrated that isotonic saline injections at the NEPs of the SAN and DSN within the SCM and SM muscles are an effective and safe treatment for alleviating pain in the posterior neck, trapezius, and interscapular areas associated with nerve entrapment, without any complications. Patients receiving these injections experienced significant reductions in both VAS and PPID scores, indicating meaningful pain relief without any recorded complications. The findings support the growing evidence that isotonic saline injections can target muscle tension and relieve pain linked to nerve entrapment.

Seong previously defined NEPs as locations where a muscle physically compresses a nerve, leading to muscle tension, entrapment of vasa nervorum, focal ischemia, nerve membrane hyperexcitability, and the generation of various pain types [11]. With repeated microtrauma, the muscle becomes tense and tender, further entrapping or compressing the adjacent vasa nervorum, ultimately causing focal ischemia [11]. This cascade of events may induce membrane hyperexcitability, where an overly excited nerve has the potential to generate abnormal excitation signals, resulting in a range of pain types [11]. In line with the “Tong-sa hypothesis”, this study hypothesized that relieving tension in specific regions of the SCM and SM muscles, identified as potential entrapment sites for the SAN and DSN, could reduce pain by decreasing membrane hyperexcitability [11,23,24,25,26,27].

In this study, the upper third of the SCM and the mid-portion of the SM were identified as potential entrapment sites for the SAN and DSN [12,13]. The entrapment of SAN and DSN caused ischemic neural pain in the posterior neck, trapezius, and interscapular area [11]. Therefore, releasing the tension within the SCM and SM, where NEPs had formed, was hypothesized to alleviate pain in these regions. The injection therapy involved intramuscular injection of isotonic saline into the muscles causing pain, targeting the identified NEPs responsible for excessive muscle tension and potentially reducing the hyperexcitability of the specific nerves entrapped by the muscles [11,25].

While our findings highlight the importance of targeting NEPs, the therapeutic effect of isotonic saline injections may also involve additional mechanisms. Beyond releasing muscle tension, isotonic saline injections are believed to reduce pain through neuromodulation. This neuromodulatory effect can be inferred from studies [28] in neuropathic pain rat models, where saline administration demonstrated analgesic effects attributed to its neuromodulatory properties. Additionally, isotonic saline injections are thought to exert biochemical changes by modulating local inflammation. A previous study on ultrasound-guided saline injections showed that these injections alleviate pain by inhibiting cytokines and inflammatory mediators, thereby reducing local inflammation [29]. Moreover, the mechanical effects of the injections may decompress the vasa nervorum, improving blood flow and reducing ischemic pain. These potential mechanisms underscore the standalone therapeutic value of isotonic saline injections, even in the absence of adjunct chemical agents.

As observed in a previous study [11], a notable aspect of this research is the finding that palpation of the identified NEPs in the upper third of the SCM and mid-portion of the SM muscles elicited considerable tenderness during the pre-treatment phase. After treatment, the reductions in the VAS and PPID scores were accompanied by a concurrent reduction in tenderness at these NEP sites. Although not quantified in this study, the corresponding decrease in tenderness at NEP sites, proportional to the improvement in VAS and PPID scores, suggests a potential relationship. Future research should focus on systematically quantifying this phenomenon to further understand the correlation between pre-treatment tenderness at NEP sites and treatment outcomes.

Moreover, in this study, we verified the effectiveness of a new isotonic injection therapy by recording a MCID of 4.2 for neck pain on the VAS, which is significantly higher than the expected MCID of 2.6 identified in other studies [30]. This result suggested that isotonic saline injections offer a significant clinical impact on pain reduction, underscoring their effectiveness for treating neck pain. MCID is an important indicator for evaluating the effectiveness of a treatment, providing a criterion for determining whether the change experienced by a patient is clinically meaningful. Therefore, the observed increase in MCID in this study represents an important discovery that improves the quality of life for patients.

Several studies have suggested that the injection of isotonic saline into muscles for pain reduction in the control groups is comparably effective and not statistically inferior to interventions in the experimental groups. These interventions include platelet-rich plasma injections for chronic rotator cuff tears, lidocaine injections for fibromyalgia, autologous blood and corticosteroid injections for lateral epicondylitis, and prolotherapy injections for chronic lower-back pain [31,32,33,34]. The improvement in pain following the injection of isotonic saline in these studies was considered a placebo effect [31,32,33,34]. However, our findings indicate that isotonic saline itself may exert a true therapeutic effect, particularly when targeting NEPs, which warrants further investigation.

Recent studies have investigated the effects of isotonic saline for the treatment of chronic migraines, heel pain, and ulnar-sided wrist pain. In our study, we exclusively utilized isotonic saline to target NEPs without employing other commonly used chemical agents, such as lidocaine or steroids [23,25,26,27]. Regardless of the solution used, the injection of isotonic saline was found to reduce the sensitivity of the nerves, thereby relieving the compression of the vasa nervorum. Moreover, isotonic saline is safer and more readily available than other chemical agents [24]. Notably, our study is among the first to apply isotonic saline injections to address NEPs specifically within the SCM and SM muscles without additional agents like lidocaine or steroids, focusing exclusively on the physiological response to saline. The relative safety and accessibility of isotonic saline further supports its use as an effective, minimally invasive treatment option for musculoskeletal pain.

Despite our promising results, this study has several limitations. It is based on a retrospective chart review with a small sample size, and the treatment duration varied widely (2 to 97 days) among patients, complicating comparisons. Although we included an ESWT group as a control to help isolate treatment effects, no radiological or electromyographic analyses were conducted to further support clinical findings. Moreover, we lack morphological evidence, such as ultrasonographical imaging, to confirm that the pain was caused by nerve entrapment within the muscle. Therefore, future studies with larger sample sizes, consistent treatment intervals, radiographic evaluations, controlled conditions, and morphological imaging evidence are needed to substantiate and expand upon these findings. In particular, the introduction of 3D high-resolution ultrasound technology, through MR microscopy [35], will allow for the precise identification of micro-nerve entrapment sites, enabling more accurate clinical diagnosis and evaluation of treatment effectiveness.

Despite these limitations, this study importantly demonstrated the effectiveness of isotonic saline injections in alleviating posterior neck, trapezius, and interscapular pain associated with nerve entrapment, and these injections did not cause complications.

In conclusion, isotonic saline injections at NEPs within specific neck muscles, such as SCM and SM muscles, might potentially provide an effective and complication-free technique for alleviating neck pain associated with possible nerve entrapment. Accordingly, this study underscores the importance of further investigation into non-invasive treatments for musculoskeletal pain.

## Figures and Tables

**Table 1:** Baseline characteristics of patients receiving ESWT and injection treatments.

Variables	Total (n = 68)		
	Patients with ESWT (n = 34)	Patients with Injection (n = 34)	*p*-Value
**Age (years)**	44.9 ± 11.7	44.2 ± 15.2	0.824
**Sex**			
**Male, n (%)**	14 (41.2)	16 (47.1)	0.625
**Female, n (%)**	20 (58.8)	18 (52.9)	
**Duration of symptoms (days)**	135.9 ± 379.3	172.1 ± 342.5	0.681
**Initial VAS score**	5.5 ± 1.6	6.2 ± 1.3	0.058
**Initial PPID score**	100	100	-
**Duration of treatments (days)**	16.1 ± 21.2	24.1 ± 22.7	0.138
**Frequency of injections**	3.8 ± 1.9	5.0 ± 3.2	0.060

Mean ± SD. Abbreviations: ESWT, Extracorporeal Shock Wave Therapy; VAS, Visual Analogue Scale; PPID, Percent Pain Intensity Difference.

**Table 2:** Comparison of changes in VAS and PPID scores between injection and ESWT treatments.

	Total (n = 68)		
Variables	Patients with ESWT (n = 34)	Patients with Injection (n = 34)	*p*-Value ^a^
**VAS score**			
Baseline	5.5 ± 1.6	6.2 ± 1.3	0.058
Final follow-up	2.8 ± 1.9	2.0 ± 1.0	0.038
∆ _from baseline to final_	2.7 ± 2.0	4.2 ± 1.2	<0.001
*p*-value ^b^	<0.001	<0.001	
**PPID score**			
Baseline	100.0 ± 0.0	100.0 ± 0.0	-
Final follow-up	50.0 ± 29.2	33.5 ± 17.7	0.007
∆ _from baseline to final_	50.0 ± 29.2	66.5 ± 17.7	0.007
*p*-value ^b^	<0.001	<0.001	

Mean ± SD, *p*-values were calculated using a paired *t*-test. ^a^ *p*-value for comparison between ESWT and injection therapy. ^b^ *p*-value for changes before and after treatment. Abbreviations: ESWT, Extracorporeal Shock Wave Therapy; VAS, Visual Analogue Scale; PPID, Percent Pain Intensity Difference.

**Table 3:** Effect sizes of each treatment-induced change in VAS and PPID scores before and after treatment.

Variables	Mean Difference	Standard Deviation of Difference	*t*-Value	Degrees of Freedom (DF)	Effect Size (Cohen’s *d*)	*p*-Value
**VAS score difference**						
Patients with ESWT (n = 34)	2.706	1.962	8.040	33	1.379	<0.001
Patients with injection (n = 34)	4.177	1.186	20.530	33	3.521	<0.001
**PPID score difference**						
Patients with ESWT (n = 34)	50.000	29.233	9.970	33	1.710	<0.001
Patients with injection (n = 34)	66.471	17.732	21.860	33	3.521	<0.001

Abbreviations: ESWT, Extracorporeal Shock Wave Therapy; VAS, Visual Analogue Scale; PPID, Percent Pain Intensity Difference.

## Data Availability

The original contributions presented in this study are included in the article. Further inquiries can be directed to the corresponding authors.
